# Utilizing Peptide Ligand GPCRs to Image and Treat Pancreatic Cancer

**DOI:** 10.3390/biomedicines6020065

**Published:** 2018-06-02

**Authors:** Gail L. Matters, John F. Harms

**Affiliations:** 1 Department of Biochemistry and Molecular Biology, The Pennsylvania State University College of Medicine, Hershey, PA 17033, USA; 2 Department of Biological Sciences, Messiah College, Mechanicsburg, PA 17055, USA; jharms@messiah.edu

**Keywords:** G protein–coupled receptors, cholecystokinin, gastrin, gastrin-releasing peptide, bombesin, neurokinin, neurotensin, somatostatin

## Abstract

It is estimated that early detection of pancreatic ductal adenocarcinoma (PDAC) could increase long-term patient survival by as much as 30% to 40% (Seufferlein, T. et al., *Nat. Rev. Gastroenterol. Hepatol.* **2016**, *13*, 74–75). There is an unmet need for reagents that can reliably identify early cancerous or precancerous lesions through various imaging modalities or could be employed to deliver anticancer treatments specifically to tumor cells. However, to date, many PDAC tumor-targeting strategies lack selectivity and are unable to discriminate between tumor and nontumor cells, causing off-target effects or unclear diagnoses. Although a variety of approaches have been taken to identify tumor-targeting reagents that can effectively direct therapeutics or imaging agents to cancer cells (Liu, D. et al., *J. Controlled Release* **2015**, *219*, 632–643), translating these reagents into clinical practice has been limited, and it remains an area open to new methodologies and reagents (O’Connor, J.P. et al., *Nat. Rev. Clin. Oncol*. **2017**, *14*, 169–186). G protein–coupled receptors (GPCRs), which are key target proteins for drug discovery and comprise a large proportion of currently marketed therapeutics, hold significant promise for tumor imaging and targeted treatment, particularly for pancreatic cancer.

## 1. Introduction

**Figure 1:**
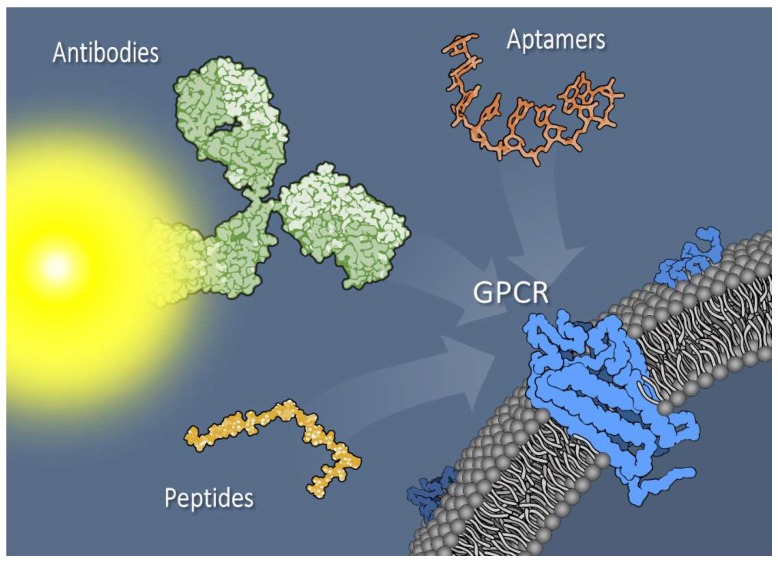
Pancreatic tumor cell surface membrane with G protein–coupled receptors (GPCRs) can be targeted with a variety of reagents, including antibodies (depicted as dye-conjugated) or antibody fragments, aptamers, or small peptides. Additionally, novel bi- or multivalent combinations of targeting agents exhibit promise as tools for imaging and treatment. Targeting agents are not drawn to scale.

The utility of reagents to enhance tumor imaging or direct treatment often relies on tumor-targeting ligands that bind to proteins that are overexpressed on the surface of malignant cells [1,2,3]. Tumor-directed targeting can make use of antibodies, peptides, small molecules, or other moieties, and can result in a higher cargo concentration either within or on the surface of tumor cells than would be attained without targeting [4]. In pancreatic ductal adenocarcinoma (PDAC), development of targeted therapies has focused on receptor tyrosine kinases (RTKs) or their downstream pathways, with limited efficacy [5]. G protein-coupled receptors (GPCRs) represent an opportunity to develop new targeted therapeutics and imaging agents for pancreatic cancer [6] (Figure 1).

## 2. G Protein-Coupled Receptors

GPCRs are plasma membrane proteins composed of seven transmembrane-spanning α-helices linked by three intracellular and three extracellular loop regions, an extracellular amino-terminal domain, and an intracellular carboxyl-terminal domain. Classical GPCR signaling is initiated by a ligand interacting with extracellular receptor loop/transmembrane domain residues, which form a ligand-binding pocket. This interaction triggers a conformational change in the receptor that initiates binding and activation of intracellular heterotrimeric G proteins. The exchange of guanosine diphosphate (GDP) for guanosine triphosphate (GTP) on the G alpha subunit dissociates G alpha from the G beta/gamma subunits and activates numerous downstream effector pathways [7,8]. Receptor activation is followed by desensitization and internalization. Once activated, GPCRs are phosphorylated by G protein kinases (GPKs), and cytosolic β-arrestins can then bind to the GPCRs, competing with the GPCR-G protein interaction and downregulating G protein-mediated signaling. The GPCR/β-arrestin complex can follow one of the endocytic pathways [9], in which GPCRs can either be recycled back to the plasma membrane or sent to the lysosomes for degradation [10].

GPCRs play an important role in cancer progression, and these proteins have been utilized as therapeutic and imaging targets. Since many chemotherapeutic agents are only active intracellularly, transmembrane transport of targeted cargos is a key issue. Unlike single transmembrane spanning proteins, which are often cleaved by proteases such as matrix metalloproteases (MMPs) to release their ectodomains [11,12], ligand-induced GPCR internalization improves intracellular bioavailability of the cargo. GPCR recycling also provides cell membrane–associated targets for additional rounds of internalization. Increased expression and activity of GPCRs is evident at all stages of PDAC tumor development, and GPCRs contribute to tumor cell proliferation, tumor progression through stimulation of angiogenic and metastatic cascades, and the creation of a proinflammatory tumor microenvironment and evasion of immune cell recognition [13].

Recent evidence suggests that mutations in GPCRs and their associated G proteins are common in tumors—approximately 20% of all cancers contain mutated GPCRs or G alpha subunits [14]. For example, defects that impact GPCR trafficking can contribute to receptor retention at the cell surface and altered downstream signaling. Activating mutations in GPCR-associated proteins, particularly *GNAS*, which encodes the Gs-alpha subunit, can be present in up to 12% of pancreatic tumors [10,14]. Reduced GTPase activity leads to constitutive signaling that can drive tumor progression. In addition, crosstalk between GPCR and RTK signaling pathways can stimulate receptor transactivation and has been linked to oncogenic Kras activation in early-stage PDAC [15,16].

GPCRs mediate a broad range of autocrine and paracrine responses in cancer cells. They bind to a diverse group of ligands, including small peptides (e.g., gastrointestinal hormones), lipids (e.g., sphingosine-1-phosphate, prostaglandins), and proteins (e.g., chemokines) [8]. The density of GPCRs on the cell surface is typically 10^3^–10^4^ receptors/cell, which should be adequate to ensure ample uptake of the targeted drug cargo or to bind sufficient imaging reagents to achieve quality images [17,18]. Herein, we focus on the peptide hormone–ligand subfamily of GPCRs and their use in developing reagents to identify and treat pancreatic cancer.

## 3. CCKRs

The peptides gastrin and cholecystokinin (CCK) activate two structurally related G protein-coupled receptors, the CCK1 receptor (CCK1R) and CCK2 receptor (CCK2R), which are expressed by many PDAC tumors [19]. Although highly homologous, with 50% overall identity, these receptors differ in their ligand-binding specificities and inhibitor profiles [20]. CCK1R binds with high affinity to CCK-8 amide with a sulfated tyrosine. CCK2R binds gastrin and CCK with similar affinity and does not discriminate between sulfated and nonsulfated CCK, as binding is directed by the final four amino acids of these peptides (Trp-Met-Asp-Phe-NH_2_, although leucine or norleucine can be interchanged for methionine to improve stability without altering binding affinity) [21]. Functionally, CCK2R expressed on pancreatic tumors plays a role in tumor cell proliferation and angiogenesis [22].

Beginning in the late 1990s, many groups explored the use of CCK1R and CCK2R for tumor imaging and treatment. While anti-CCK2R antibodies have been developed [23,24], most targeting reagents have been peptide analogs of either CCK or gastrin. With a radionuclide chelator attached to the N-terminus of CCK or gastrin peptide analogs, a variety of reagents have been created for tumor imaging and radiotherapy, including CCK-8, gastrin 10, mini-gastrin, gastrin dimers, and cyclic gastrin analogs [25,26,27,28,29,30,31]. Although tumor uptake relative to other tissues was good, the ability of the peptide-targeted constructs to deliver cargo to tumors was limited by high proteolytic turnover in serum, often with less than 10% of the reagent remaining in circulation 10 minutes post-injection [32]. One approach to extending the half-life of gastrin- or CCK-based reagents has been to inhibit the activity of the protease responsible for gastrin/CCK degradation, neutral endopeptidase (NEP). Co-injection of the NEP inhibitor phosphoramidon with gastrin analogs increased their half-life in circulation and improved tumor uptake [33,34,35]. Finally, nanoparticles can be bioconjugated with gastrin peptide to improve tumor-specific uptake. Attaching gastrin 10 to fluorescent dye–loaded calcium phosphosilicate nanoparticles enhanced particle uptake by orthotopic pancreatic tumors in a murine model [36].

## 4. GRP/Bombesin Receptors

This family of peptide receptors contains gastrin-releasing peptide receptor (GRPR), neuromedin B receptor (NMBR), and bombesin receptor subtype 3 (BRS3), which are overexpressed by a number of cancers, including PDAC [37]. PDAC cells have previously been targeted with a GRPR ligand radiolabeled for positron emission tomography (PET) imaging, or conjugated with Gd^3+^ for magnetic resonance imaging (MRI) [38,39]. Human gastrin releasing peptide (GRP) as well as mammalian bombesin (BN), which differ by only 1 out of 10 amino acids, have been utilized for GRP- or BN-drug conjugates with paclitaxel or docetaxel. Compared to free drug, the peptide-drug conjugates resulted in enhanced cytotoxicity in vitro [40,41,42]. However, the efficacy of these compounds against pancreatic tumors in vivo remains unclear [43].

## 5. Neurokinin Receptors

The neurokinin-1 receptor (NK1R) and its peptide ligand, substance P (SP), regulate many tumor cell processes, including proliferation, angiogenesis, migration, invasion, and metastasis [44]. NK1R is upregulated in human pancreatic tumors, especially in advanced tumors with poor prognosis, and has recently been implicated in perineural invasion of PDAC tumors [45]. In mice, a subpopulation of PanIN epithelial cells express NK1R. Evidence suggests that these are acinar cell–derived neoplastic PanIN epithelial cells, opening the potential for using NK1R-targeted imaging for detection of early lesions [46]. Tumor imaging using an NK1R-targeted fluorescent dye has been used during surgery to facilitate identification and resection of NK1R-positive lesions [47,48]. In PET imaging, ^64^Cu-NK1R-NOTA is a promising reagent for identifying NK1R-expressing tumors [49], and NK1R-targeted cytotoxic drugs are also under development [50].

## 6. Neurotensin Receptors

Neurotensin receptor (NTS1) has been identified on several PDAC cells lines, in human PDAC tissues, and in late-stage PanINs and PDAC liver metastases, with lower expression in chronic pancreatitis [51,52]. NTS1 binding of the ligand neurotensin (NT) activates mitogenic signaling, while a selective NTS1 antagonist, SR 48692, reduces PDAC cell proliferation [53,54]. Because NT interacts with the NTS1 receptor with high affinity and only the six C-terminal amino acids of NT are required for receptor binding, bioconjugation of NT peptide to a variety of reagents holds potential for improving their delivery to NTS1-expressing tumors [17]. Biodistribution studies using NT-targeted probes in PDAC tumor–bearing mice showed high tumor-specific uptake of ^68^Ga-labeled NT peptides in vivo [55]. In addition to NT peptides, NTS1 small-molecule antagonists labeled with ^18^F and ^177^Lu also demonstrated tumor cell internalization and retention in vivo with low kidney and liver uptake [56,57]. Liposomes functionalized with a branched neurotensin peptide, NT4, and loaded with doxorubicin have been assessed for antitumor cell efficacy in vitro [58].

Interestingly, recent evidence shows that there is crosstalk between the insulin/IGF-1 receptor and NT/NTS1 signaling pathways, which leads to activation of the oncogenic YAP/TAZ pathway. Stimulation of PDAC cells with both insulin and neurotensin results in nuclear localization of YAP, decreased YAP phosphorylation, and increased expression of YAP/TEAD-regulated genes, while treatment with either insulin or neurotensin alone only modestly induced the expression of these genes [59]. This suggests that either antagonism of NTS1 or blockade of downstream signaling pathways connecting to YAP could a be promising therapeutic target for PDAC [60].

## 7. Somatostatin Receptors

Somatostatin receptor (SSTR) subtypes SSTR2, SSTR3, and SSTR5 are present in human PDAC tumors based on mRNA expression [61,62]. The short half-life of somatostatin (SST) prompted the development of several peptide analogs for therapeutic and imaging purposes, the most clinically relevant of which is octreotide (OCT). This eight-amino-acid peptide binds to SSTR2 with high affinity and triggers receptor endocytosis [18,63,64]. OCT-drug conjugates, created by direct coupling of camptothecin or paclitaxel to the N-terminus of the peptide, were cytotoxic to cancer cell lines that overexpressed SSTR2 [65] and induced regression of subcutaneous CFPAC-1 tumors in athymic mice [66]. More recently, a reagent that combined MRI/optical imaging capability and a synthetic peptide (PTR86) with high affinity for somatostatin receptors showed efficient imaging and targeting of pancreatic tumors [67]. An SST analog dual-labeled with a radionuclide and fluorescent dye has recently been evaluated in a preclinical colon cancer model system [68].

Interestingly, a PDAC-specific interaction between two GPCRs, the mu opioid receptor (MOR) and SSTR2, has recently been identified [69]. The presence of this GPCR heterodimer correlated with increased oncogenic signaling and tumor progression and antagonists to either receptor triggered heterodimer internalization. This suggests that the MOR-SSTR2 heterodimer may represent a unique PDAC-specific target. Investigation of other novel GPCR heterodimers in PDAC may uncover new opportunities for therapeutic targeting with higher tumor cell specificity.

## 8. Dual-Targeted Agents

A challenge for the development of tumor-targeted drug delivery or imaging is the level at which the target protein is expressed. It is well documented that PDAC tumors and metastatic lesions are heterogeneous with regard to their expression of GPCRs and other cell-surface receptors [70]. Dual-targeted reagents are capable of targeting different GPCRs simultaneously, or a GPCR and another extracellular protein. These reagents can achieve better specificity than targeting the proteins individually. Dual-targeting agents also can provide better sensitivity through a greater number of potential tumor cell binding sites, thus enabling clearer visualization of cancerous lesions or improved drug delivery [71].

Simultaneous targeting of two independent GPCRs was achieved using a peptide that combined ligands for the CCK2 receptor and the melanocortin 1 receptor (also known as MC1R) [72]. This bivalent reagent joins seven amino acids from melanocortin to the CCK-4 tetrapeptide via a synthetic fluorescently tagged linker. In vitro, the hybrid ligand was able to bind both cell-surface receptors, demonstrating a 12-fold higher specificity for cells expressing both receptors. The ability of the bivalent ligand to improve the imaging of tumors in vivo was confirmed using tumor cell lines engineered to express either the MSH receptor, CCK2 receptor, or both.

Dual targeting can also exploit a target protein on a nonmalignant cell type within the tumor microenvironment (TME) in addition to a tumor-cell GPCR. Demonstrating this strategy, bombesin was fused to an RGD peptide motif, thereby targeting both a GPCR and integrin αvβ3 on tumor endothelial cells [73]. The resulting ^68^Ga-labeled heterodimeric peptide has been successfully employed in PET imaging. A second example, while not targeting a GPCR, demonstrates the utility of bivalent targeting. Pancreatic tumor xenografts were imaged using a heterodimer of antibody fragments targeting CD105 on the tumor vasculature and tissue factor (TF) on tumor cells [74,75]. Further explorations of multivalent combinations of a GPCR-targeted ligand with other TME targets would constitute novel advancements.

## 9. Nonpeptide Targeting to GPCRs: Aptamers

RNA and DNA aptamers are single-stranded, structured oligonucleotides that have promise for both targeted tumor imaging and drug delivery while avoiding some of the common disadvantages of peptide and antibody targeting [76]. Targeting with antibodies can be associated with a risk of inappropriate immune response, while peptides are typically susceptible to proteolytic degradation in the systemic circulation, making them unsuitable for many in vivo applications. Small molecules such as antagonists, while having a well-defined chemical structure and good stability, can have low target selectivity or rapid clearance in vivo [3]. Aptamers have a reproducible structure and can be easily modified to resist nucleases, can be synthesized at a lower cost, are stable to changes in temperature and pH, and can refold spontaneously once conditions normalize. They have fewer nonspecific interactions in the systemic circulation, are less immunogenic, and display high binding affinity to targets with dissociation constant (Kd) values in the nanomolar range. Due to their low molecular weight (25–70 nucleotides is equivalent to 8–20 kDa), aptamers can also penetrate tumor tissues more efficiently than antibodies or Fab fragments [77,78].

Aptamers can be attached to a variety of payloads, including small interfering RNAs (siRNAs), cytotoxic drugs, or nanoparticles, which improves the selective delivery and efficacy of the cargo [79,80,81,82]. Tumor-targeting aptamers have been selected for cell-adhesion molecules such as EpCAM, tyrosine kinase receptors, mucins, and other cell-surface proteins [83]. For example, EGFR-targeted aptamers conjugated to gold nanospheres have been successfully used to image head and neck tumors [84]. Aptamer-based imaging agents and aptamer-targeted therapeutics are now moving into clinical trials [85]. 

Our research team has identified and characterized aptamers against the GPCR CCK2R. Using a SELEX-based library selection protocol, we selected aptamers that bound to both a synthetic peptide contained within the extracellular N-terminal domain of the CCK2R and PDAC cells expressing CCK2R in its native conformation. Negative selection with non–CCK2R-expressing cells ruled out nonspecific interactors. Overall, we identified a pool of >100 high-affinity DNA aptamers that specifically recognized the extracellular N terminus of the human CCK2R [86]. Quantitatively, we have shown that one of the selected CCK2R aptamers (AP1153) has a 300-fold higher affinity for CCK2R than its native peptide ligand, gastrin. As evidence for its utility as a pancreatic tumor targeting agent, we demonstrated that AP1153 was internalized by PDAC cells in a receptor-mediated fashion, and that bioconjugation of AP1153 to the surface of fluorescent nanoparticles enhanced whole-animal optical detection of PDAC tumors in vivo. Others have identified aptamers that bind to the GPCR NTS1, although their further development for diagnostic or therapeutic use has not yet been shown [87].

Finally, aptamers can have direct therapeutic benefit as antitumor reagents. A variety of RNA aptamers that bind to β2-adrenoceptor (β2AR), a non–peptide-liganded GPCR, have been shown to stabilize this receptor in active, inactive, or ligand-specific conformations [88]. Similar to neutralizing antibodies, aptamers can block the interaction between ligand and receptor. Although not directed toward a GPCR, anti–PD-L1 aptamers block the PD-1/PD-L1 signaling axis, reducing tumor growth and improving immune surveillance [89]. An anti–CTLA-4 aptamer has also shown promise for delivering siRNA cargo. The lack of therapeutic efficacy of siRNA-mediated gene silencing is due in part to low siRNA internalization by tumor cells [90]. Conjugation of an anti–CTLA-4 aptamer to a STAT3 siRNA helped to overcome this limitation and achieved STAT3 gene silencing in both tumor-associated T cells and tumor cells [91,92]. GPCR-binding aptamers that disrupt ligand-receptor interactions and abrogate downstream GPCR signaling, or that improve delivery of either siRNAs or drug-loaded nanoparticles to tumors, could have application as new PDAC therapeutics [76].

## 10. Conclusions

Earlier detection and targeted therapies for pancreatic cancer will undoubtedly improve patient survival [93,94]. However, developing reagents capable of specifically targeting tumors for imaging and drug delivery remains a significant challenge. GPCRs represent a class of tumor cell surface proteins with well-characterized ligands, well-understood pathways for internalization and recycling, and well-documented signaling capabilities, including crosstalk with other oncogenic signaling pathways. Identifying and validating GPCR-specific imaging or therapeutic reagents could provide new tools to make clinically significant improvements in PDAC patient care and achieve the goal of improving survival rates for patients battling this disease.
